# Bilateral multicenter pseudohemangiomatous interstitial hyperplasia of the breast: a case report

**DOI:** 10.1080/23320885.2023.2193273

**Published:** 2023-03-24

**Authors:** Chengcai Yao, Changchun Liu

**Affiliations:** Department of Breast Surgery, Sixth Affiliated Hospital of South China University of Technology, and Sixth Clinical College of South China University of Technology, Foshan, P.R. China

**Keywords:** Pseudohemangiomatous interstitial hyperplasia, mammary gland, breast reconstruction, case report

## Abstract

Bilateral multicenter breast pseudohemangiomatous stromal hyperplasia (PASH) is a rare, benign breast disease. Here, we report on a female patient with bilateral multicenter PASH who underwent a mastectomy and prosthetic reconstruction. The surgery was successful, and no recurrence was observed during the 18 months of follow up.

## Introduction

Pseudoangiomatous stromal hyperplasia (PASH) is a benign breast disease defined microscopically by the proliferation of mammary stroma. It often presents clinically as an incidental finding during the evaluation of other benign or malignant lesions, or less commonly as a palpable, well-circumscribed breast mass [1,2]. Imaging studies are unable to distinguish PASH from other benign tumors such as fibroadenomas or phyllodes tumors [3]. Hence, the final diagnosis of PASH relies on histopathology and immunohistochemistry (IHC) [4]. Local mass removal is a common surgical procedure but recurrence can occur, especially in patients with multifocal PASH [5]. The clinical manifestations of PASH include a tumor-forming lesion (tumorous PASH) or gigantomastia (diffuse PASH). Most of the cases addressed in the past were unilateral tumour PASH, and very few cases of bilateral PASH have been recorded [5,6]. In this article, we describe a rare case of bilateral multicenter tumorous PASH in a patient that underwent total mastectomy and one-stage implant-based breast reconstruction. The patient was then observed for 18 months with very good results in terms of tumor recurrence and breast appearance.

## Case report

The patient was a 43-year-old premenopausal woman. She was admitted to the hospital with bilateral breast enlargement, swelling, and pain for two months. Two months prior, she felt a rapid enlargement of both breasts without apparent provocation, accompanied by swelling and pain. Nine days prior, she felt worsening swelling and pain in both breasts, mainly in the right breast. In addition, she had breast asymmetry, with the right side significantly larger than the left. After an initial examination, the outpatient physician enrolled her in our department for further diagnosis and treatment. The patient had a history of hypertension for 11 years and cesarean section surgery. She was taking irbesartan–hydrochlorothiazide and amlodipine besylate, and her blood pressure was normal.

**Figure 1.**
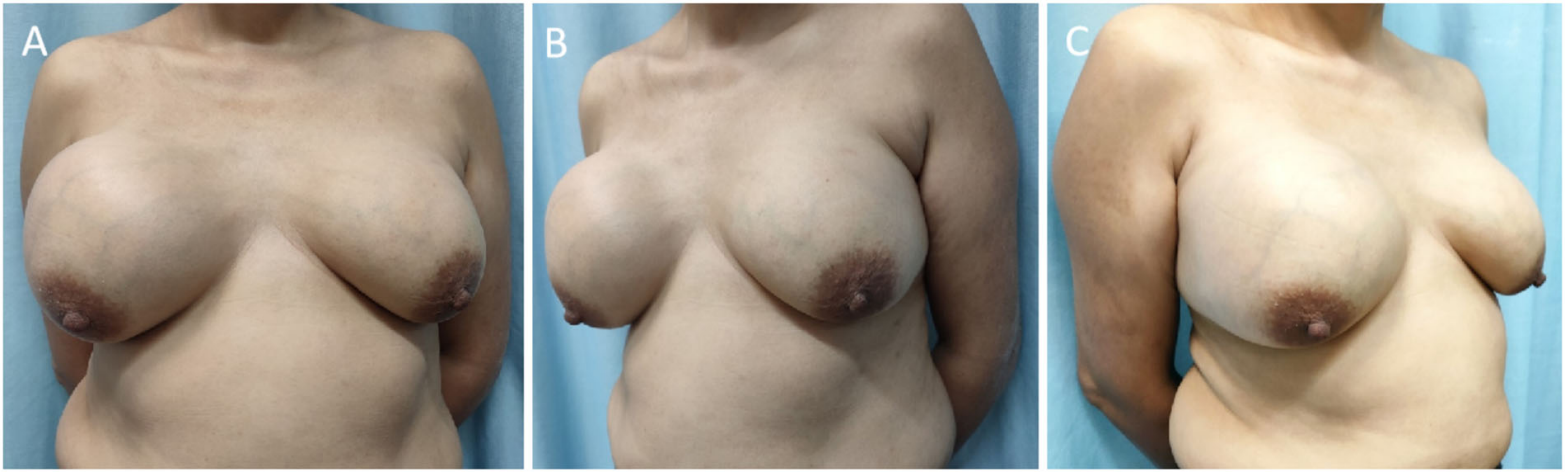
PASH patient before surgery. (A – C) Pre-surgery photographs taken from different angles, indicating breast asymmetry. The right breast was significantly larger than the left, and mild venous dilatation can be observed on the surface of both breasts. PASH, Pseudoangiomatous stromal hyperplasia.

**Figure 2.**
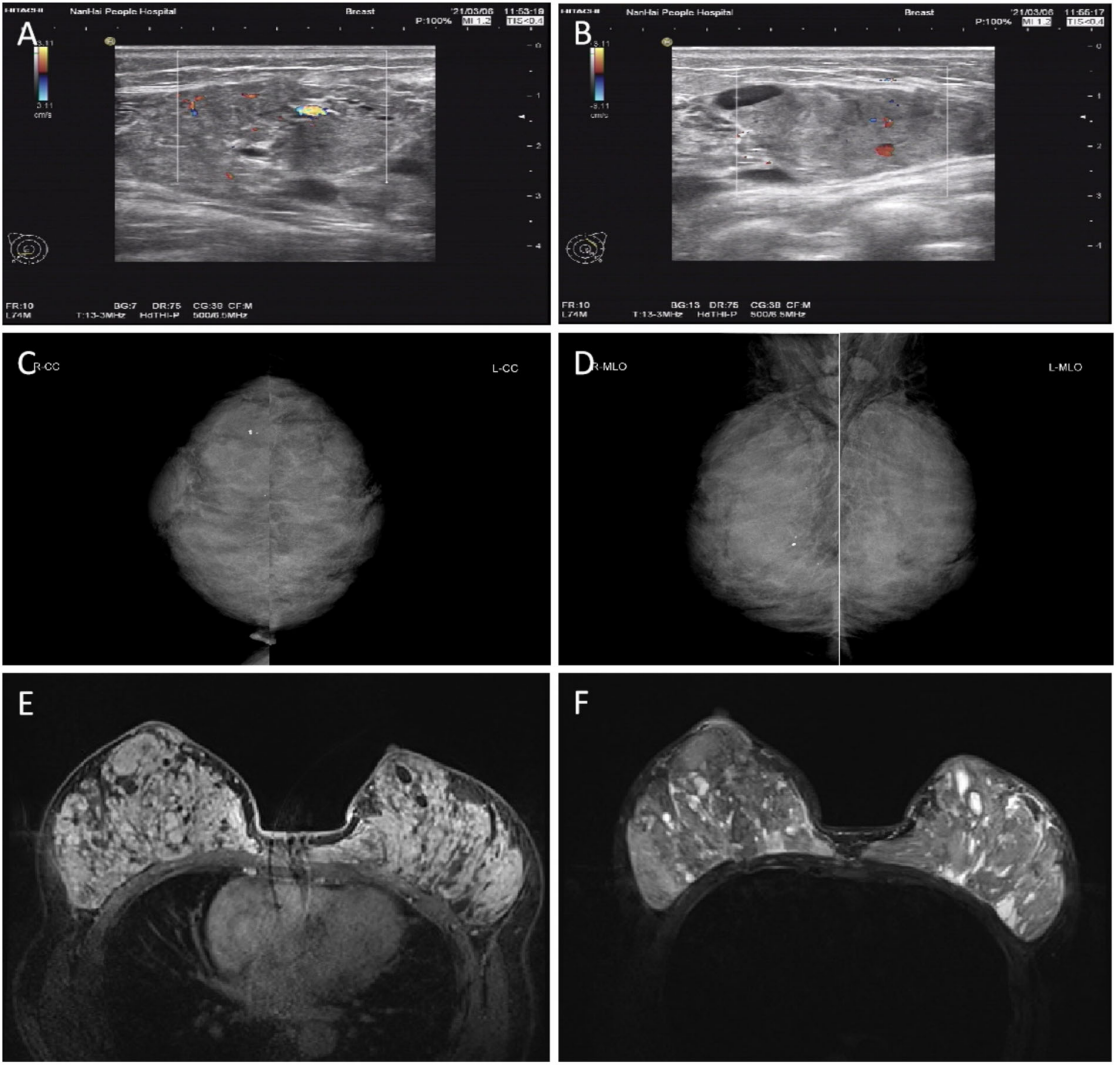
Preoperative color ultrasonography (A, B), mammography (C, D), and MRI (E, F) of bilateral breasts. Color ultrasonography (A, B) of the breast indicated a solid mass with a clear boundary, a low blood flow signal (A), and cystic changes. Mammography (C, D) indicated dense and lamellar bodies of both mammary glands, with no obvious nodular sensation, bilateral axillary lymph node shadows, and a few coarse calcifications in the right mammary gland. MRI (E, F) indicated dense glands in both breasts, with multiple mixed long T1 (E) and long T2 (F2) abnormal signal shadows in each quadrant. MRI, Magnetic resonance imaging.

Upon specialist physical examination, the patient’s breasts were found to be asymmetrical, with the right breast significantly larger than the left. Mild venous dilatation was observed on the surface of both breasts (Figure 1(A–C)). Multiple solid masses of different sizes were palpable in both breasts, ranging in diameter from 1.5 cm × 1.5 cm to 6.0 cm × 6.5 cm, with tough quality, clear boundaries, and a smooth surface. The masses did not adhere to the skin and were moveable, with mild tenderness. No enlarged lymph nodes were palpable in the bilateral axilla or the upper and lower clavicle. Preoperative color ultrasound, mammogram, and magnetic resonance imaging (MRI) results of the breasts are presented in Figure 2.

**Figure 3.**
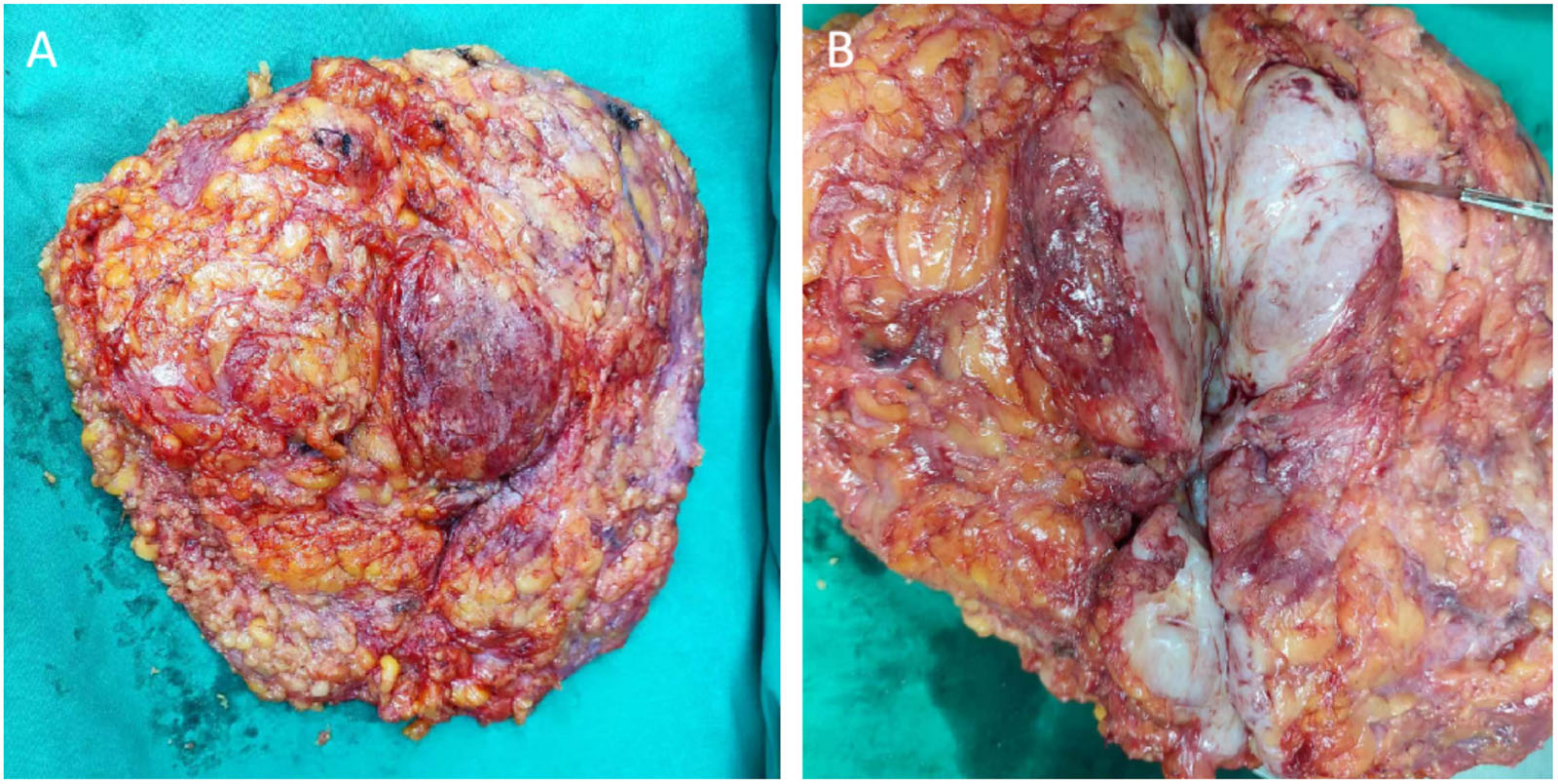
The gross specimen of the left breast excised during the surgery. Multiple tumors of different sizes can be observed (A). When the tumors were cut open, their surface was gray and fresh (B).

**Figure 4.**
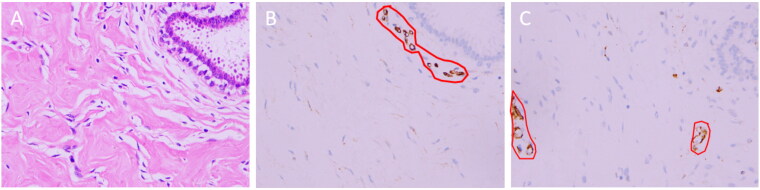
Postoperative pathology of the breast. Microscopically, the mammary stromal cells proliferated and formed overlapping fissures. Spindle cells were attached, they were mild in shape, with no obvious atypia, and no mitotic image of the nucleus was found (A, hematoxylin and eosin (H&E) staining, ×400). Immunohistochemistry (IHC): Interstitial cells were positive for CD34 staining (Figure E, streptavidin-perosidase (SP) conjugated method, ×400) and negative for CD31 staining (Figure F, streptavidin-perosidase (SP) conjugated method, ×400). CD34 was expressed in the cytoplasm (shown in brown), and the nucleus is shown in blue. However, the vascular endothelial cells were positive for CD31 staining, which is expressed in the cytoplasm, shown in brown (red-circled areas in B and C). IHC, Immunohistochemistry.

**Figure 5.**
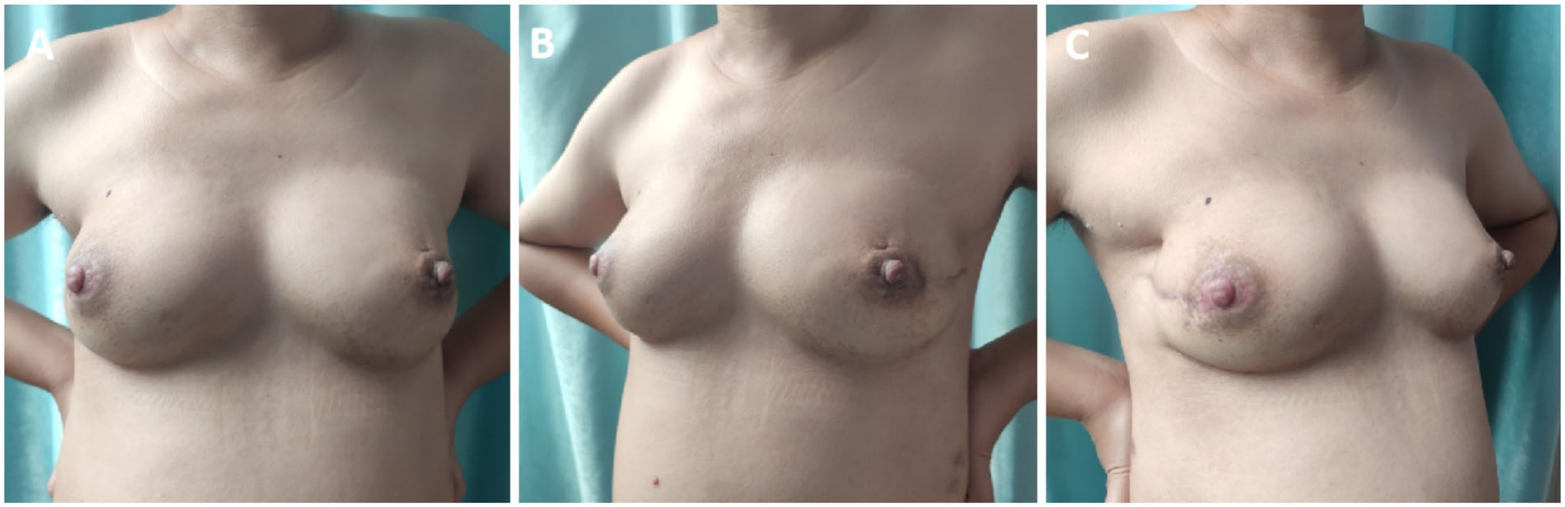
Follow-up 18 months after the operation. There is no tumor recurrence, the reconstructed breast was symmetrical, and the shape was excellent. (A–C) Post-surgery photographs taken from different angles.

After admission, a core needle biopsy was performed, which resulted in a diagnosis of PASH. Because of the rapid growth of the tumors and their spread throughout the breasts, the patient and her family feared malignancy. Therefore, the patient chose to undergo a bilateral mastectomy and one-stage implant-based breast reconstruction. Nipple-areolar complex-sparing mastectomy and one-stage silicone gel implant-based breast reconstruction were performed on 15 April 2021 at the request of the patient and her family. During the surgery, incisions were created at the 3 o‘clock (left breast) and 9 o‘clock (right breast) positions. After subcutaneous mastectomy, the lower margin of the pectoralis major muscle was separated, and the upper part of a titanium-coated polypropylene mesh (specifications, 235 mm × 135 mm × 160 mm; model, ultralightweight, large size, ref. 6000638. PFM Medical, Cologne, Germany) and the lower margin of the pectoralis major muscle were sutured using 3-0 Vicryl line (Vicryl, Ethicon, Norderstedt, Germany). Subsequently, the breast prosthesis (CPG 321, Catalog # 354-1258, Volume:315cc. Johnson & Johnson (China) Medical Equipment Co., Ltd.) was placed below the pouch to completely wrap the prosthesis. Finally, one drainage tube was attached to the inner and outer sides of the chest wall with a negative pressure bottle, and bilateral subpectoral immediate prosthesis combined with titanium-coated polypropylene mesh breast reconstruction was completed. During the surgery, it was observed that masses covered most of the breast tissue. When the masses were cut open, the sections were pale and tender (Figure 3). The surgery was successful, and the patient recovered and was discharged from the hospital seven days post-surgery. PASH was further confirmed by postoperative histopathology and immunohistochemistry (IHC) (Figure 4). In the first year after the surgery, color ultrasound and physical examination of the breasts were performed by a physician every three months. After one year, follow up was changed to every six months. Breast magnetic resonance imaging (MRI) was performed 12 months after the surgery. Follow-up tests at 18 months indicated no recurrence of the tumors. According to the standard of the Joint Center for Radiation Therapy [7], the reconstructed breasts were symmetrical in appearance and had excellent shape (Figure 5), and the patient was satisfied with the results.

## Discussion

PASH is a benign, proliferative mesenchymal lesion of the breast first described by Vuitch et al. [8]. It is a condition characterized by hyperplasia of stromal myofibroblasts. PASH tissue strongly resembles angiogenic proliferation upon histological analysis, hence the term “pseudoangiomatous.”

The etiology and pathogenesis of PASH has remained unknown. Studies have found that certain types of drug resistance to antiepileptic drugs and hormone replacement therapy may be related to PASH [9,10]. In this case, because the patient had an 11-year history of hypertension, she was on long-term regular medication for this condition (irbesartan–hydrochlorothiazide and amlodipine besylate). Whether these drugs cause PASH has not yet been determined.

In clinical practice, most cases of PASH are incidental histological findings in breast biopsy specimens or surgical resection specimens. For example, Ibrahim et al. [1] demonstrated that 23% of 200 histological samples of various malignant and benign tumors contained PASH. Interestingly, simple PASH rarely presents as a palpable lump [11–13]. PASH can be classified as tumorous PASH or diffuse PASH, based on clinical presentation. Most lesions in diffuse PASH are not accessible, whereas masses in neoplastic PASH are palpable during physical examination. Tumorous PASH typically presents as a single mass or multiple localized, ductile, mobile masses and is often misdiagnosed as fibroadenoma or phyllodes [4]. Furthermore, PASH presents clinically by possibly causing breast asymmetry in unilateral cases [14], or rarely in gigantomastia in bilateral cases [15]. In this paper, we describe a 43-year-old postmenopausal female patient with multiple, localized, ductal, mobile masses. Due to the rapid growth of the right breast lump, the right breast had a larger volume than the left breast, and the two sides of the breast were asymmetrical. The outpatient physician misdiagnosed the condition as multiple fibroadenomas or phyllotumor. This is in general agreement with what has been recorded in the literature [4].

In mammography, PASH appears similar to fibroadenoma and is generally considered a noncalcified, well-defined mass. In ultrasonography, PASH usually appears as an oval, well-defined, hypoechoic mass. In magnetic resonance imaging, PASH normally has progressive enhancement (T1), and high-signal slit-like spaces can be observed on T2-weighted and short τ inversion recovery images [16]. However, some scholars believe that the current magnetic resonance imaging method is not suitable for making an exact diagnosis of PASH without a histological diagnosis [17]. Therefore, the final diagnosis of PASH must be confirmed by pathology. However, the limited tissue volume of fine-needle aspiration cytology may produce cell-free specimens, and core needle biopsy is currently recommended [16,18]. In this patient, the multiple bilateral breast masses were found to be like multiple fibroadenomas upon breast ultrasound, mammography, and MRI. The final diagnosis was confirmed by core needle biopsy pathology.

Microscopically, PASH of the breast is histologically characterized by anastomoses and collagenous stroma with slit-like spaces and is lined by flattened, spindle-shaped cells. These clear spaces, which may mimic microscopic vascular channels, do not contain red blood cells. Sometimes, for an accurate diagnosis, an IHC examination is also needed. The mesenchymal cells of PASH express vimentin, CD34 (an endothelial cell marker), actin, desmin, calponin, and progesterone receptor, but not CD31 (vascular marker) or factor VIII (endothelial cell marker) [5,12,19]. In this case, the patient had an enlargement of the collagen fibers in the breasts, in which many fissures were formed and coincided with each other. The center of the cleft spindle cells had a mild morphology and negligible heterozygosity, and no nuclear division was observed. Mesenchymal cells tested positive for CD34 but not CD31; however, the peripheral vascular tissue tested positive for CD31. Therefore, the combination of morphology and IHC in this PASH diagnosis was well established.

Because PASH is a benign disease of the breast and is not considered a precancerous lesion or risk factor for cancer, most specialists recommend extensive local resection as the preferred treatment [20]. However, total mastectomy can be considered for extensive lesions that have spread throughout the breast [21]. There are few reports on one-stage prosthetic reconstruction performed after resection [4,6]. Here, we discuss a 43-year-old female patient with bilateral multicenter PASH. As requested by the patient and her family, nipple-areolar complex-sparing mastectomy and one-stage breast prosthesis reconstruction were performed. A follow-up of 18 months following surgery indicated no signs of tumor recurrence, the shape of the reconstructed breasts was excellent, and the patient was extremely satisfied.

## Conclusions

In summary, PASH is a rare, benign disease caused by hyperplasia of mesenchymal cells in the breast. Breast imaging is not specific to this disease, and the final diagnosis relies on histopathology and IHC. Local resection is an effective first option for PASH treatment, but there is a risk of recurrence. For bilateral multicenter PASH, total mastectomy and one-stage breast reconstruction are promising options.
